# Plasma rich in growth factors versus Mitomycin C in photorefractive keratectomy

**DOI:** 10.1097/MD.0000000000024139

**Published:** 2021-01-22

**Authors:** Ronald M. Sanchez-Avila, Edmar E. Uribe-Badillo, Javier Fernández-Vega Sanz, Francisco Muruzabal, Nancy Jurado, Belén Alfonso-Bartolozzi, Jose F. Alfonso, Begoña Baamonde, Eduardo Anitua, Jesus Merayo-Lloves

**Affiliations:** a Instituto Universitario Fernández-Vega, Fundación de Investigación Oftalmológica, Universidad de Oviedo; b Biotechnology Institute (BTI), Vitoria, Spain; c Universidad de San Martin de Porres, Lima, Peru; d University Institute for Regenerative Medicine and Oral Implantology (UIRMI), Vitoria, Spain.

**Keywords:** platelet-rich plasma, plasma rich in growth factors, photorefractive keratectomy, refractive surgery

## Abstract

To evaluate the efficacy and safety of plasma rich in growth factors (PRGF) in photorefractive keratectomy (PRK) versus Mitomycin C (MMC).

This is a comparative, longitudinal and retrospective case-control study (MMC vs PRGF), in patients with a spherical correction from −0.25 to −8.00 D and cylinder correction from −0.25 to −3.00. The uncorrected distance visual acuity (UDVA), refractive efficacy and safety indices, and changes in endothelial cell density were evaluated. The predictability was assessed with the postoperative manifest spherical equivalent.

Forty-four patients (72 eyes) were treated with MMC and twenty-five patients (45 eyes) with PRGF. The final UDVA (LogMar) in MMC was 0.029 ± 0.065 and in PRGF it was 0.028 ± 0.048 (p = 0.383). The efficacy index for MMC was 0.98 ± 0.10 and 1.10 ± 0.46 for patients treated with PRGF (p = 0.062). The safety index for MMC was 1.03 ± 0.11 and 1.12 ± 0.46 (p = 0.158) for PRGF group. The change percentage of endothelial cell density was 0.9 ± 11.6 for MMC and 4.3 ± 13.1 for PRGF (p = 0.593). The predictability for MMC was 92.1% and for the PRGF was 91.9% (p = 0.976). Hyperemia, eye pain and superficial keratitis were observed in 11.1% of the MMC group; no adverse events were observed with the PRGF.

The use of PRGF in PRK surgery is as effective as MMC. The PRGF shows a better safety profile than MMC for its intraoperative use in PRK.

## 1 Introduction

Photorefractive keratectomy (PRK) is a corneal surgical procedure increasingly performed in the West in the last decade. It has become a fast, reliable and very safe procedure, with visual acuity results similar in time and visual quality as LASIK.^[1]^ It is essential to know the results in efficacy, predictability and safety, to evaluate a refractive surgical technique.^[2]^ However, the corneal haze is a risk associated with the surgical procedure, which can lead to reduced visual quality and alterations in the refractive results, and it may be necessary to perform keratoplasties in severe cases.^[3]^ This corneal opacity can generally appear within the first 12 weeks from the surgery; however, there are some cases of late appearance after 2 years.^[4]^ Topical corticosteroids are used to resolve these corneal opacities in a few weeks; however, there are cases with no-resolution, generating discomfort and distress in the patient.^[3]^

Since 2000, Majmudar et al, described for the first time their results using the Mitomycin C (MMC) at 0.02% for 2 minutes.^[5]^ In this PRK surgery study, a decrease in the corneal haze was observed in patients treated with MMC. These results increased the percentage of ophthalmologists who adopted the use of MMC in PRK surgery to prevent the haze appearance. However, the use of MMC has some risks, 1 of the most important being the possibility of inducing endothelial damage when it is introduced into the anterior chamber due to its antimetabolite effect. There is a direct relationship between the time of contact with MMC and the harmful effect on the corneal endothelium so that the higher the thickness of the ablation, the greater the amount of MMC used and the higher the possibilities of inducing endothelial damage.^[6,7]^

The plasma rich in growth factors (PRGF) is a blood derivative product of autologous origin that is used for the treatment of several ocular surface and cornea diseases such as dry eye, persistent epithelial defects, corneal ulcers, banded keratopathies, high-risk keratoplasties, among others.^[8,9]^ PRGF formulations have essential features for tissue regeneration such as their cellular trophic effect, antimicrobial, antifibrotic, and anti-inflammatory properties.^[9–11]^ PRGF eye drops treatment reduced the haze formation after PRK surgery in an animal model; therefore, it could be a viable alternative for the prevention of corneal stromal opacity after PRK in the clinical practice.

The present study evaluated the clinical findings of PRGF compared to the application of MMC, for the improvement of visual acuity, refractive efficacy, predictability and safety. The safety of both treatments was also evaluated, including the prevention of haze formation after PRK surgery.

## 2 Material and methods

### 2.1 Study design

A retrospective, longitudinal, observational, descriptive, and comparative study was conducted including patients who underwent PRK surgery at the Instituto Universitario Fernández-Vega between February 2013 and October 2016. Informed consent was obtained from all patients included in the study for the use of PRGF and the surgical procedure. Ethical approval was obtained from the Institutional Clinical Research Committee for the use of autologous PRGF in patients. This study was performed following all the principles of the Declaration of Helsinki.

This study was designed to compare the efficacy and safety of the use of intra-operative PRGF eye drops in PRK surgery in comparison to MMC application. Patients were divided into 2 groups: those who received the standard intraoperative MMC treatment (control group) and those who received the PRGF therapy (study group). Data were obtained retrospectively from medical records, and patients were required to have a minimum follow-up of 6 months after surgery.

### 2.2 Patients

Patients who were selected for the study should be over 18 years of age, maintain stable refraction for more than 1 year (defined as a change of no more than 0.50 D in sphere or cylinder), corneal topography within normality, central corneal thickness greater than 420 μm, spherical correction range of −0.25 to −8.00 D, and cylinder correction range of −0.25 to −3.00. Also, patients had to stop wearing soft contact lenses for at least 2 weeks and hard contact lenses for at least four weeks to be evaluated as candidates for PRK surgery. Patients with degenerative corneal diseases, refractive instability, trauma or previous corneal surgery, glaucoma, degenerative retinal diseases, diabetes mellitus, keratoconus, systemic vascular diseases, or active ocular infections were excluded from the study.

Demographic data of patients (age, sex), their complete history of ophthalmological examination including uncorrected-distance visual acuity, corrected distance visual acuity (CDVA), refraction measurements (cylinder and sphere), manifest spherical equivalent (MSE), slit-lamp biomicroscopy, intraocular pressure measured with a Perkins tonometer, computerized corneal topography, corneal pachymetry, endothelial cell density (ECD), and pupil diameter were obtained from the medical records.

### 2.3 PRGF preparation

PRGF eye drops were performed according to the manufacturer protocol (BTI, Biotechnology Institute, S.L., Miñano, Álava, Spain),^[9]^ and the Endoret-PRGF ophthalmology kit was used. Briefly, peripheral blood was collected into 9-mL tubes with 3.8% sodium citrate as an anticoagulant. It was centrifuged at 580 g for 8 min at room temperature in an Endoret System centrifuge (BTI Biotechnology Institute, S.L., Miñano, Álava, Spain), and the whole PRGF column was collected after centrifugation, avoiding the collection of the layer containing the leukocytes using the closed Endoret-PRGF system (BTI Biotechnology Institute, S.L., Miñano, Álava, Spain). PRGF eye drops were incubated at 37°C for 1 hour. The plasma supernatants were filtered, aliquoted and taken to the surgery room to be used, the rest of the PRGF was used topically, at a dose of 1 drop every 6 hours for 6 weeks. PRGF maintains its stability at room temperature for 72 hour. PRGF can be stored at low temperatures (−18 / −20°C) to maintain its stability for 3 months.

### 2.4 Surgical technique

All patients were rinsed topically with povidone-iodine 10%, and topical anaesthesia with tetracaine/oxybuprocaine (4 mg/ml, 1 mg/ml, Alcon Healthcare SA, Spain) was applied. An 8 mm zone was marked using a trephine to establish the area to be deepithelialized, the de-epithelialization was performed with a rotating brush (Amoils, Innovative Excimer Solutions, Toronto, Ontario, Canada), and photoablation was performed with an Excimer laser (STAR S4 IR Excimer Laser, Abbott Laboratories Inc, IL). The ablation diameter did not exceed 8.0 mm in any case. Then, the topical application of mitomycin C 0.02% in the stromal bed was performed for 10 seconds per treated diopter, up to a maximum of 40 seconds (control group). Finally, the eye surface and cornea were rinsed with 30 mL of Balanced Salt Solution (BSS, Carl Zeiss Meditec AG, Jena, Germany). In the case of patients treated with PRGF, this was applied on a water-insoluble spongy lamina (Espongostan, Takeda Pharmaceutical, Spain) and was kept in contact with the stromal bed for 1 minute (study group), no subsequent rinse with BSS. At the end of the procedure, both groups were treated with 0.2% hyaluronic acid eye drops and with a bandage contact lens.

All patients were evaluated daily until corneal re-epithelialization was achieved, then bandage contact lens was removed, and the follow-up visits were carried out at 2 weeks, 1, 3, and 6 months after surgery. All patients were postoperatively treated with moxifloxacin 0.5% (Vigamox; Alcoon Surgical, Inc.) 4 times per day for 1 week, fluorometholone 0.1% (4 times a day for the first 4 weeks, after that, 3 times a day for 2 more weeks, then twice-daily for other 2 weeks, and finally once a day for 2weeks), bromfenac 0.9 mg/mL (twice-daily for 1 week).

### 2.5 Outcome measures

To compare the efficacy of PRGF and MMC after PRK surgery several outcome variables were collected, including the refractive efficacy through postoperative UDVA, the safety with the maintenance of post-surgical CDVA, predictability with postoperative MSE and stability with changes in MSE over time. The visual acuity (UDVA, CDVA) was measured with the Snellen optotype and transformed to LogMAR. Objective refractions, keratometries and pupillometries were measured with a Kerato/Refractometer KR-1W (Topcon, Barcelona, Spain). Central corneal thickness measurement was performed with ultrasonic pachymeter (DGH 5100 contact pachymeter, DHG Technology Inc, Exton, PA; OcuScan RXP, Alcon Laboratories, Inc, Fort Worth, TX). The ECD was analyzed in an automated way with a specular microscope (SP-1P, Topcon, Barcelona, Spain) before surgery and at the end of the follow-up. Any adverse events or complications showed along the patient follow-up period, were recorded to assess the safety of both treatments, including corneal haze (grade 0 to 4).^[12]^

### 2.6 Statistical analysis

The statistical analysis was performed using SPSS v15.0 statistical software for Windows (SPSS Inc., Chicago, IL). Descriptive statistics were carried out using absolute and relative frequency distributions for qualitative variables, and mean values and standard deviations analyses for the quantitative variables. The normal distribution of variables was confirmed with the Kolmogorov-Smirnov and Shapiro-Wilk tests. The Mann-Whitney *U* test was used to evaluate differences between treatment groups. The difference before and after treatment was analyzed using the Wilcoxon nonparametric statistical test for nonparametric variables, and *t*-student for parametric variables. The chi-square test was used to compare the categorical variables. The Spearman test was used for correlation analyses. The statistical significance level was established at *P* < .05.

## 3 Results

Sixty-nine patients with a total of 117 affected eyes were included and evaluated in the present study. Forty-eight patients (69.6%) were operated bilaterally, and 21 (30.4%) unilaterally. Forty-four patients (72 eyes, 61.5%) received treatment with MMC, while 25 patients (45 eyes, 38.5%) were treated with PRGF eye drops. Twenty-eight patients (40.6%) were treated bilaterally in the MMC group, and 20 patients (29.0%) in the PRGF group. Most of the patients were women (n = 39, 56.5%) with a total number of 66 (56.4%) treated eyes, of which 39 eyes (33.3%) were included into the MMC group and 27 (23.1%) into PRGF group. The number of eyes treated in men was 51 (43.6%), of which 33 eyes (28.2%) were treated with MMC and 18 (15.4%) with PRGF. The initial CDVA was different between both treatment groups (*P* = .023) with worse initial visual acuity in the PRGF group. Flat keratometry was similar in both treatment groups (*P* > .05); however, curved keratometry was higher in the PRGF group (*P* = .029). There are no differences between the groups for the sphere, cylinder and MSE variables (Table 1). No statistical differences (*P* > .05) were found in photoablation, stromal bed, re-epithelialization time, final UDVA and follow-up time variables between both treatment groups (Table 2).

**Table 1:** Pre-operative baseline characteristics by treatment group.

Parameters, *mean ±* *SD (range)*	MMC	PRGF	*P-*Value
Age (years)	33.8 ± 13.3 (19.0–74.0)	37.6 ± 15.5 (22.0–68.0)	.911
Sex,			
*Female (%)*	24 (34.8)	15 (21.7)	.536
*Male (%)*	20 (29.0)	10 (14.5)	
Sphere (D)	−1.75 ± 0.80 (−0.50 to −3.50)	−1.65 ± 0.71 (−0.25 to −3.50)	.675
Cylinder (D)	−0.56 ± 0.37 (0.0 to −1.75)	−0.58 ± 0.58 (0.0 to −3.00)	.669
MSE (D)	−2.03 ± 0.76 (−0.75 to −3.63)	−1.95 ± 0.75 (−0.75 to −3.75)	.586
CDVA (logMAR)	0.013 ± 0.040 (0.000 to 0.222)	0.046 ± 0.115 (0.000 to 0.523)	.023^∗^
CCT (μm)	529.5 ± 39.8 (435.0 to 618.0)	536.4 ± 42.9 (423.0 to 627.0)	.225
Flat Keratometry (D)	43.7 ± 1.3 (41.0 to 46.5)	44.2 ± 1.8 (41.0 to 49.0)	.114
Curve Keratometry (D)	44.2 ± 1.4 (41.0 to 47.5)	45.0 ± 2.1 (41.0 to 50.8)	.029^∗^
Pupil (mm)	6.5 ± 1.0 (3.5 to 8.0)	6.4 ± 1.0 (4.5 to 8.0)	.670
IOP (mm Hg)	12.9 ± 2.0 (9.0 to 19.0)	13.0 ± 1.8 (9.0 to 16.0)	.482
ECD (cells/mm^2^)	2689.6 ± 318.8 (1758.0 to 3350.0)	2671.3 ± 519.1 (1159.0 to 3707.0)	.580

**Table 2:** Surgery and follow-up parameters.

Parameters, *mean ±* *SD (range)*	MMC	PRGF	*P*-Value
Photoablation (μm)	29.2 ± 13.0 (5.0–55.0)	29.5 ± 12.0 (8.0–58.0)	.924
Surgical stromal bed (μm)	487.8 ± 45.3 (389.0–640.0)	497.2 ± 43.1 (401.0–578.0)	.183
Re-epithelialization time (hours)	63.3 ± 12.6 (36.0–72.0)	64.3 ± 11.8 (36.0–72.0)	.722
36h, n (%)	4 (5.6)	1 (2.2)	
48h, n (%)	20 (27.8)	13 (28.9)	
72h, n (%)	48 (66.7)	31 (68.9)	
UDVA (logMAR) final	0.029 ± 0.065 (0.000–0.301)	0.028 ± 0.048 (0.000–0.155)	.383
Follow-up time (mo)	17.7 ± 5.5 (10.2–26.6)	16.6 ± 5.3 (11.7–28.6)	.398

The analyses of the different variables evaluated in the study before and after both treatments showed that CDVA (LogMAR) was reduced significantly (*P* = .01) after treatment with MMC while no significant differences (p = 0.102) were observed in PRGF group. On the other hand, a statistically significant improvement (p < 0.05) was obtained in the sphere, cylinder, MSE, flat, and curve keratometry after both treatments. However, no significant changes were found in ECD before and after treatment with MMC or PRGF (Tables 3 and 4).

**Table 3:** Outcome variables analyzed in the PRGF group.

Parameters, *mean ±* *SD (range)*	Baseline	Final	*P*-Value
CDVA (logMAR)	0.046 ± 0.115 (0.000 to 0.523)	0.021 ± 0.041 (0.000 to 0.115)	.102
Sphere (D)	−1.65 ± 0.71 (−3.50 to −0.25)	−0.10 ± 0.23 (−1.00 to 0.0)	< .001^∗^
Cylinder (D)	−0.58 ± 0.58 (−3.00 to 0.00)	−0.12 ± 0.26 (−1.00 to 0.00)	< .001^∗^
MSE (D)	−1.95 ± 0.75 (−0.75 to −3.75)	−0.16 ± 0.31 (0.00 to −1.38)	< .001^∗^
Flat Keratometry (D)	44.2 ± 1.8 (41.0 to 49.0)	42.6 ± 1.6 (40.0 to 47.0)	< .001^∗^
Curve Keratometry (D)	45.0 ± 2.1 (41.0 to 50.8)	43.4 ± 1.9 (41.0 to 48.5)	< .001^∗^
ECD (cells/mm^2^)	2671.3 ± 519.1 (1159.0 to 3707.0)	2740.1 ± 452.8 (1494.0 to 3433.0)	.308

**Table 4:** Outcome variables analyzed in the MMC group.

Parameters, *mean ±* *SD (range)*	Baseline	Final	*P*-Value
CDVA (logMAR)	0.013 ± 0.039 (0.000 to 0.222)	0.001 ± 0.001 (0.000 to 0.005)	.01^∗^
Sphere (D)	−1.75 ± 0.80 (−3.50 to −0.50)	0.01 ± 0.23 (−0.75 to 1.00)	< .001^∗^
Cylinder (D)	−0.56 ± 0.37 (−1.75 to 0.00)	−0.10 ± 0.30 (−1.00 to 0.50)	< .001^∗^
MSE (D)	−2.03 ± 0.76 (−0.75 to −.63)	−0.04 ± 0.31 (1.25 to −1.00)	< .001^∗^
Flat Keratometry (D)	43.7 ± 1.3 (41.0 to 46.5)	41.8 ± 1.7 (37.8 to 44.8)	< .001^∗^
Curve Keratometry (D)	44.2 ± 1.4 (41.0 to 47.5)	42.2 ± 1.7 (38.3 to 45.5)	< .001^∗^
ECD (cells/mm^2^)	2689.6 ± 318.8 (1758.0 to 3350.0)	2685.7 ± 334.3 (1821.0 to 3390.0)	.622

Besides, percentage changes obtained before and after follow-up for each outcome variable were compared between both treatment groups (MMC and PRGF) (Table 5). In summary, the percentage change for CDVA was 3.4 ± 11.1 in treatment with MMC and 11.5 ± 45.9 after PRGF treatment, showing no significant differences (*P* = .332) between both groups. Moreover, no significant differences (p > 0.05) were observed in the sphere, cylinder, flat and curved keratometry and MSE variables between the treatment groups.

**Table 5:** Comparative analysis of the percentage change for each variable analyzed in both treatment groups.

Parameters, *mean ±* *SD (range)*	MMC	PRGF	*P*-Value
CDVA (logMAR)	3.4 ± 11.1 (0.0 to 66.7)	11.5 ± 45.9 (−10.0 to 200)	.332
Sphere (D)	101.2 ± 23.4 (50.0 to 250.0)	90.6 ± 33.8 (−100.0 to 100.0)	.067
Cylinder (D)	85.5 ± 53.2 (−100.0 to 200.0)	71.9 ± 53.0 (−100.0 to 100.0)	.257
MSE (D)	110.7 ± 58.8 (18.2 to 290.0)	109.9 ± 77.3 (23.5 to 428.6)	.580
Flat Keratometry (D)	−4.4 ± 2.0 (−9.1 to 0.6)	−4.0 ± 1.8 (−7.6 to −1.1)	.339
Curve Keratometry (D)	−4.4 ± 2.3 (−9.6 to 1.2)	−3.9 ± 2.2 (−8.6 to 1.7)	.210
Efficacy index	0.98 ± 0.10 (0.70 to 1.20)	1.10 ± 0.46 (0.88 to 3.00)	.062
Safety index	1.03 ± 0.11 (1.00 to 1.67)	1.12 ± 0.46 (0.90 to 3.00)	.158
ECD (cells/mm^2^)	0.9 ± 11.6 (−22.5 to 37.6)	4.3 ± 13.1 (−18.8 to 36.0)	.593

**Figure 1:**
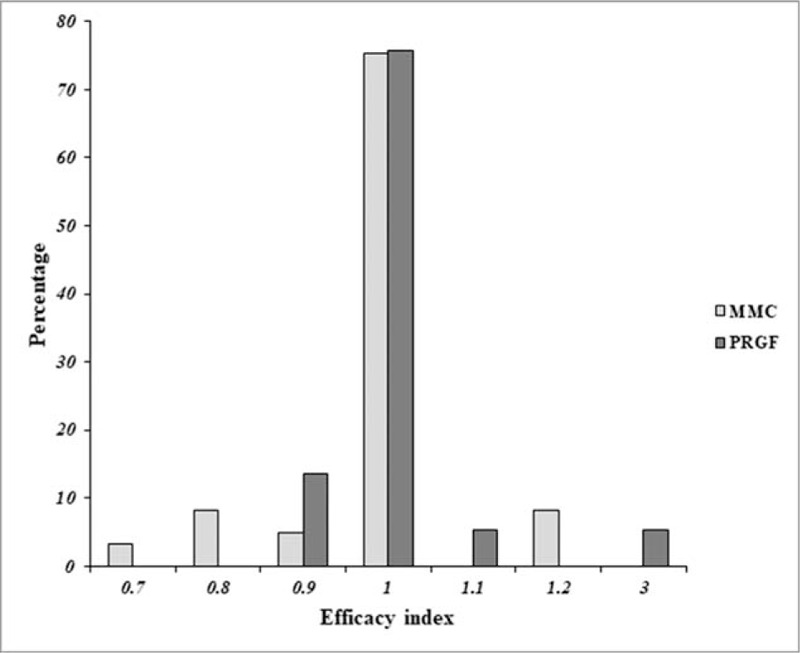
Efficacy Index by treatment group. MMC = mitomycin C, PRGF = plasma Rich in Growth Factors.

On the other hand, a statistical improvement trend (*P* = .062) was observed in the effectiveness index after PRGF treatment (1.10 ± 0.46) in comparison to the MMC group (0.98 ± 0.10). Safety index was also slightly higher in the PRGF group than in the MMC group (Table 5). When the effectiveness index was widely analyzed, the results showed that the values in the MMC group were 0.7 (3.3%), 0.8 (8.1%), 0.9 (4.9%), 1.0 (75.4%), and 1.2 (8.2%), while they were 0.9 (13.5%), 1.0 (75.7%), 1.1 (5.4%), and 3.0 (5.4%) for the PRGF group (Fig. 1). PRGF treatment showed a non-significant (*P* = .593) increase in the change percentage of ECD (4.3 ± 13.1) in comparison to the MMC group (0.9 ± 11.6) (Table 5). No reoperation was necessary for any of the treatment groups during the follow-up period.

**Figure 2:**
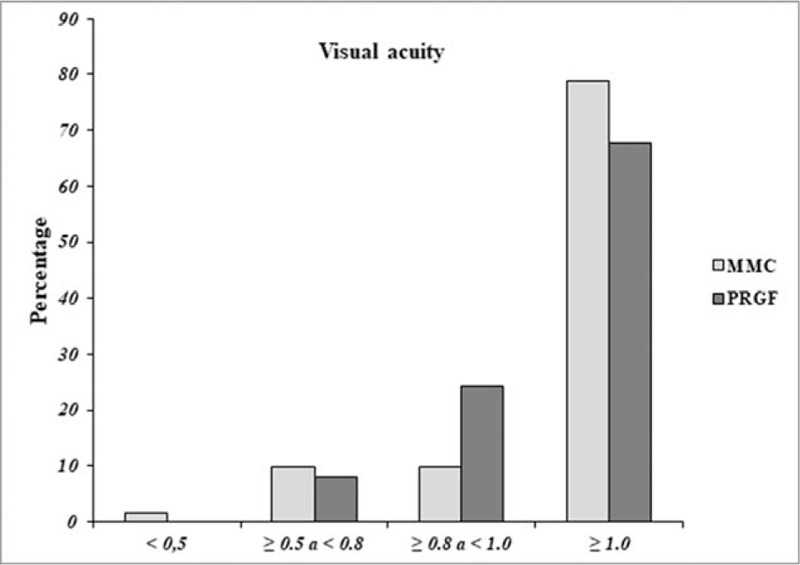
Final uncorrected visual acuity (UDVA) by treatment group. Visual acuity (decimals), MMC = mitomycin C, PRGF = plasma Rich in Growth Factors.

Predictability, defined as the percentage of eyes that were within ± 0.5D of residual MSE with respect to emmetropia at 6 months after surgery, showed no differences (*P* = .976) between both groups of treatment, showing values of 92.1% (58 eyes) for the MMC group and of 91.9% (34 eyes) for the PRGF group. The analysis of post-treatment UDVA (decimal) for the MMC group was 1.6% (< 0.5; n = 1), 9.8% (from ≥ 0.5 to < 0.8; n = 6), 9.8% (from ≥ 0.8 to <1.0; n = 6), and 78.7% (≥ 1.0; n = 48), while for the PRGF group was 0.0% (< 0.5; n = 0), 8.1% (≥ 0.5 to < 0.8; n = 3), 24.3% (≥ 0.8 to <1.0; n = 9), and 67.6% (≥ 1.0; n = 25) (Fig. 2). Furthermore, a positive correlation (*P* < .001) was found between the depth of photoablation and the epithelial closure time in both groups of treatment (R = 0.827 for the MMC group and R = 0.807 for the PRGF group).

Finally, some adverse events were found in patients of the MMC group. Eight eyes of the MMC group (11.1%) showed greater hyperemia and eye pain with mild superficial keratitis. These patients required the addition of dexamethasone 0.1% eye drops 4 times daily for 2 weeks to treat the symptoms. However, it is necessary to highlight that no adverse events related to the use of PRGF during the surgical procedure or along the follow-up period were reported. In general, although it was not a primary variable of the study, the corneal haze was evaluated using the slit lamp, no relevant clinical differences were found between the 2 treatment groups.

## 4 Discussion

Nowadays, the use of refractive procedures in the ocular surface is increasing in both the refractive management of healthy and altered corneas or corneas with ocular surface problems.^[13]^ Results in efficacy and predictability are essential; however, safety is a fundamental factor when choosing an adjuvant therapy (antimetabolite or regenerative) in refractive surgery. The corneal haze is a significant complication that must be prevented and treated due to its high potential to generate a line of sight loss or higher complications.^[3,14]^

The corneal haze after PRK surgery is multifactorial, and it can occur from a local inflammatory process to the irregularity of the underlying stroma. There is a predisposition to myofibroblast proliferation due to an increase in the level of several inflammatory cytokines such as TGF-β1 and IL-1, which may lead to corneal opacity. In most situations, several weeks are needed to resolve this ocular disorder, although there is a high risk of sequelae.^[15]^ The use of antimetabolites like MMC is widely used to prevent the haze formation because they are easy to prepare and very accessible for the prevention of corneal haze at a low cost.^[5,16,17]^ The MMC can induce alterations in the count and morphology endothelial.^[7]^ Furthermore, there is the possibility of systemic MMC diffusion into the bloodstream.^[18]^

In the present study, the efficacy and safety of MMC and PRGF application during the PRK surgical procedure has been compared, as well as their refractive results at 1 year of follow-up. No significant differences were found between both treatment groups in the amount of the photoablation tissue, UDVA and EDC baseline values. The Re-epithelialization time was of 100% after 3 days in both groups, finding a correlation between the amount of ablated tissue and the time to healing. Regarding visual results, no statistical difference was observed in the UDVA variable at the end of the follow-up between both procedures. Both treatment groups showed a significant improvement before and after the follow-up period for each refractive variables (sphere, cylinder and MSE).

On the other hand, no differences were found in ECD between the initial and the end values of the follow-up after the use of MMC or PRGF; however, the percentage change of ECD tended to improve after PRGF treatment regarding MMC group. The endothelial alterations described in other studies have been developed in patients subjected to a corneal stroma ablation higher than 5 D and with an exposure time higher than the carried out in the present study.^[7]^

The efficacy index was slightly higher in the PRGF group than in the MMC group, which points to a certain favorability towards the PRGF group; however, these results should be verified with future prospective clinical studies. No significant differences were observed between the 2 procedures concerning the safety index. The reoperations rate for all patients at 1 year of follow up was 0%; hence these results confirm the stability of both treatments over time. Predictability was very similar (>90%) in both treatment groups. About safety, 8 cases of corneal surface alterations that produced discomfort were found in MMC treated group. The addition of corticoid eye drops for at least 2 weeks was needed to improve the discomfort symptoms. No adverse events were found in patients treated with PRGF.

This study presents an entirely viable alternative to replace the use of MMCs in PRK surgeries, consisting in the application of a standardized growth factors concentrate (PRGF) with a high regenerative, anti-inflammatory, antimicrobial and antifibrotic capacity.^[9,19–21]^ It is essential to highlight that PRGF also has immunoregulatory and nerve regenerating properties,^[11,20,22,23]^ with great potential to avoid the corneal haze formation in patients who underwent refractive surgeries.^[3]^ Its effectiveness has also been demonstrated for the treatment of dry eye after LASIK.^[24]^ In vivo studies carried out in animal models for PRK, it has been observed that PRGF increases the proliferation of corneal epithelial cells, and reduces the number of SMA positive cells (responsible for myofibroblast differentiation), which corresponds to greater corneal transparency for evaluation by microscopy. In such a way that PRGF accelerates the regeneration of corneal tissue after PRK and diminishes the formation of haze.^[23]^

Some studies suggest the possibility of not using antimetabolites such as MMC in patients undergoing PRK surgery, since modern treatment techniques and the substantially smaller amount of ablated tissue may be enough for the prevention of corneal haze.^[25]^ This new situation may open the opportunity to focus the efforts in the treatment of other corneal alterations that remain unsolved such as dry eye,^[26]^ post-surgical corneal neurodeprivatization,^[27]^ and delayed epithelialization. PRGF eye drops therapy is presented as a new alternative to be used for the treatment of these unresolved ocular disorders. There are some limitations in this study, including the fact of being a retrospective and uncontrolled study; however, it opens the way for future multicenter studies that can confirm our results.

The results presented in this work suggest that PRGF eye drops could be used as an alternative treatment to replace the use of MMCs in PRK surgeries minimizing the undesired effects produced by MMC therapies. Also, PRGF treatment contains additional properties that promote ocular tissue regeneration, which is desirable in cases of refractive surgery.

## Acknowledgments

The authors would like to thank Virginia Cuadrado for her support with the English grammar.

## Author contributions

**Conceptualization:** Nancy Jurado, Jose F. Alfonso, Jesus Merayo-Lloves.

**Data curation:** Ronald Mauricio Sanchez-Avila, Edmar Edwin Uribe-Badillo, Francisco Muruzabal, Nancy Jurado.

**Formal analysis:** Ronald Mauricio Sanchez-Avila, Francisco Muruzabal.

**Funding acquisition:** Jose F. Alfonso, Jesus Merayo-Lloves.

**Investigation:** Ronald Mauricio Sanchez-Avila, Francisco Muruzabal.

**Methodology:** Ronald Mauricio Sanchez-Avila, Francisco Muruzabal, Nancy Jurado, Belen Alfonso-Bartolozzi.

**Project administration:** Jose F. Alfonso, Eduardo Anitua, Jesus Merayo-Lloves.

**Resources:** Edmar Edwin Uribe-Badillo, Eduardo Anitua.

**Software:** Ronald Mauricio Sanchez-Avila.

**Supervision:** Jose F. Alfonso, Begoña Baamonde, Jesus Merayo-Lloves.

**Validation:** Javier Fernandez-Vega Sanz, Belen Alfonso-Bartolozzi, Begoña Baamonde.

**Visualization:** Jose F. Alfonso.

**Writing – original draft:** Ronald Mauricio Sanchez-Avila, Francisco Muruzabal.

**Writing – review & editing:** Ronald Mauricio Sanchez-Avila, Edmar Edwin Uribe-Badillo, Javier Fernandez-Vega Sanz, Francisco Muruzabal, Nancy Jurado, Belen Alfonso-Bartolozzi, Jose F. Alfonso, Begoña Baamonde, Eduardo Anitua, Jesus Merayo-Lloves.
